# Evidence of a Louse-Borne Outbreak Involving Typhus in Douai, 1710-1712 during the War of Spanish Succession

**DOI:** 10.1371/journal.pone.0015405

**Published:** 2010-10-27

**Authors:** Tung Nguyen-Hieu, Gérard Aboudharam, Michel Signoli, Catherine Rigeade, Michel Drancourt, Didier Raoult

**Affiliations:** 1 Unité de Recherche sur les Maladies Infectieuses et Tropicales Emergentes, UMR CNRS 6236, IRD198, IFR48, Faculté de Médecine, Université de la Méditerranée, Marseille, France; 2 Unité d'anthropologie, UMR CNRS 6578, Faculté de médecine, Université de la Méditerranée, Marseille, France; Charité-Universitätsmedizin Berlin, Germany

## Abstract

**Background:**

The new field of paleomicrobiology allows past outbreaks to be identified by testing dental pulp of human remains with PCR.

**Methods:**

We identified a mass grave in Douai, France dating from the early XVIII^th^ century. This city was besieged during the European war of Spanish succession. We tested dental pulp from 1192 teeth (including 40 from Douai) by quantitative PCR (qPCR) for *R. prowazekii* and *B. quintana*. We also used ultra-sensitive suicide PCR to detect *R. prowazekii* and genotyped positive samples.

**Results and Discussion:**

In the Douai remains, we identified one case of *B. quintana* infection (by qPCR) and *R. prowazekii* (by suicide PCR) in 6/21 individuals (29%). The *R. prowazekii* was genotype B, a genotype previously found in a Spanish isolate obtained in the first part of the XX^th^ century.

**Conclusion:**

Louse-borne outbreaks were raging during the XVIII^th^ century; our results support the hypothesis that typhus was imported into Europe by Spanish soldiers from America.

## Introduction

As mentioned by Zinsser, infectious illness has killed more soldiers during war than weapons [1]. The current genetic tools make it possible in a certain number of cases to identify the probable causes of epidemics [2]. The combination of an anthropological approach that identifies burials from catastrophes with a molecular approach that makes it possible to identify the genes of bacteria in dental pulp has developed recently into the framework of a new speciality called paleomicrobiology [3]. Thanks to these elements, we found that the Justinian plague, like the great plague of the Middle Ages, was due to *Yersinia pestis* Orientalis [4]–[6]. We also identified the presence of *Bartonella quintana* in very ancient samples [7]. More recently, we showed that some of the soldiers of the Grand Army that died in Vilnius after the passage of the Bérézina river died of diseases transmitted by lice: *Bartonella quintana* and *Rickettsia prowazekii* [8].

**Figure 1:**
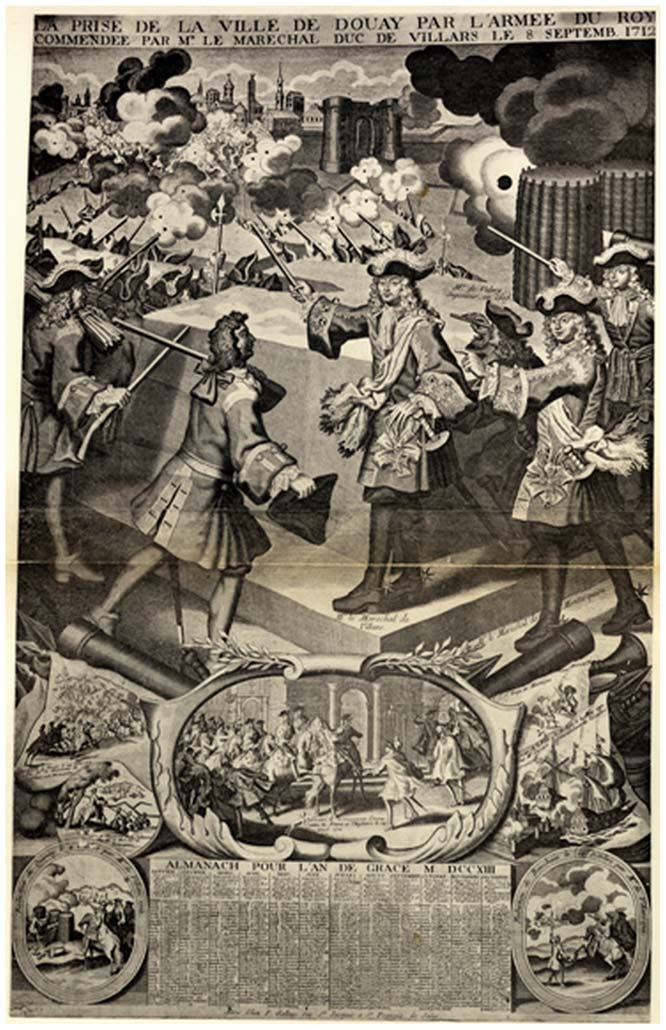
Representation of the bi siege of Douai in an Almanach.

Recently, a mass grave dating back to the 18th century was explored in Douai, France. The city of Douai was besieged from 1710 to 1712, being successively occupied by the French and then the Dutch and then retaken by French in 1712 during the war of Spanish succession (Figure 1). These events were in the framework of a generalised European war, with France and Spain opposing the other nations, the battle being carried out on the French side by Louis XIV “Le Grand Monarque.” The investigation of this mass grave showed that few skeletons presented lesions compatible with weapon wounds. This led to the assumption that a certain number of these skeletons were caused by an epidemic that occurred during the siege. Indeed, the medical condition of the men was very bad in spite of their young age. This could be consistent with an epidemic of diseases transmitted by lice.

## Materials and Methods

### Source of the materials

**Figure 2:**
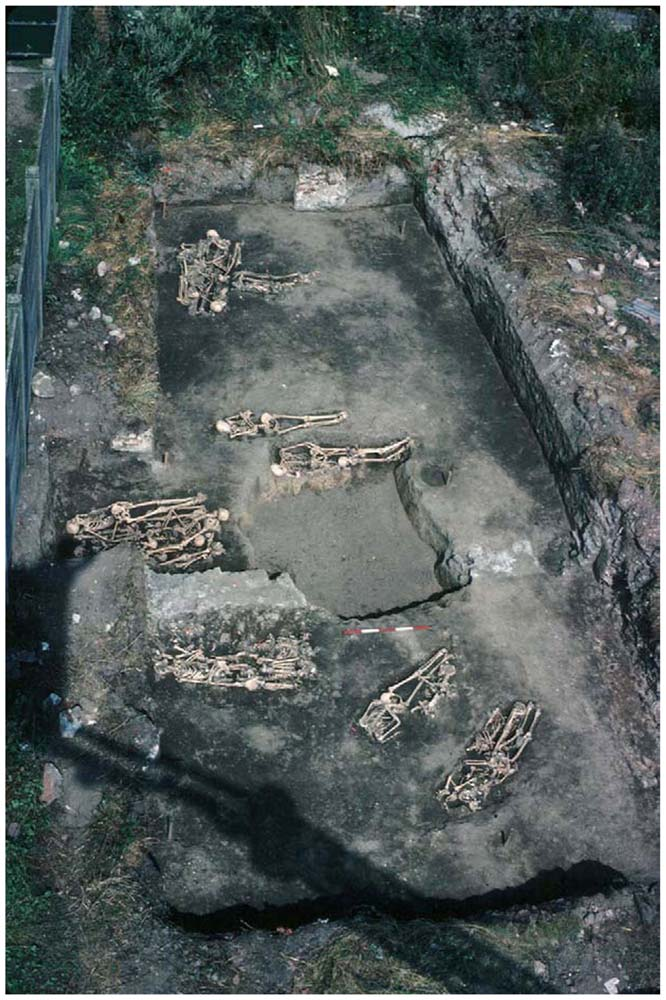
General view of the burial site of Douai.

**Figure 3:**
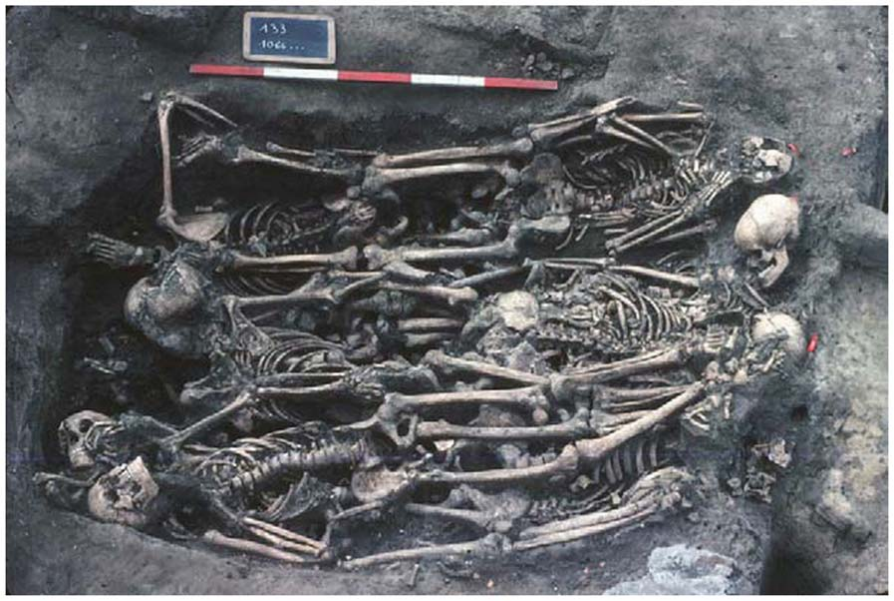
Featuring multiple burials discovered in Douai.

**Figure 4:**
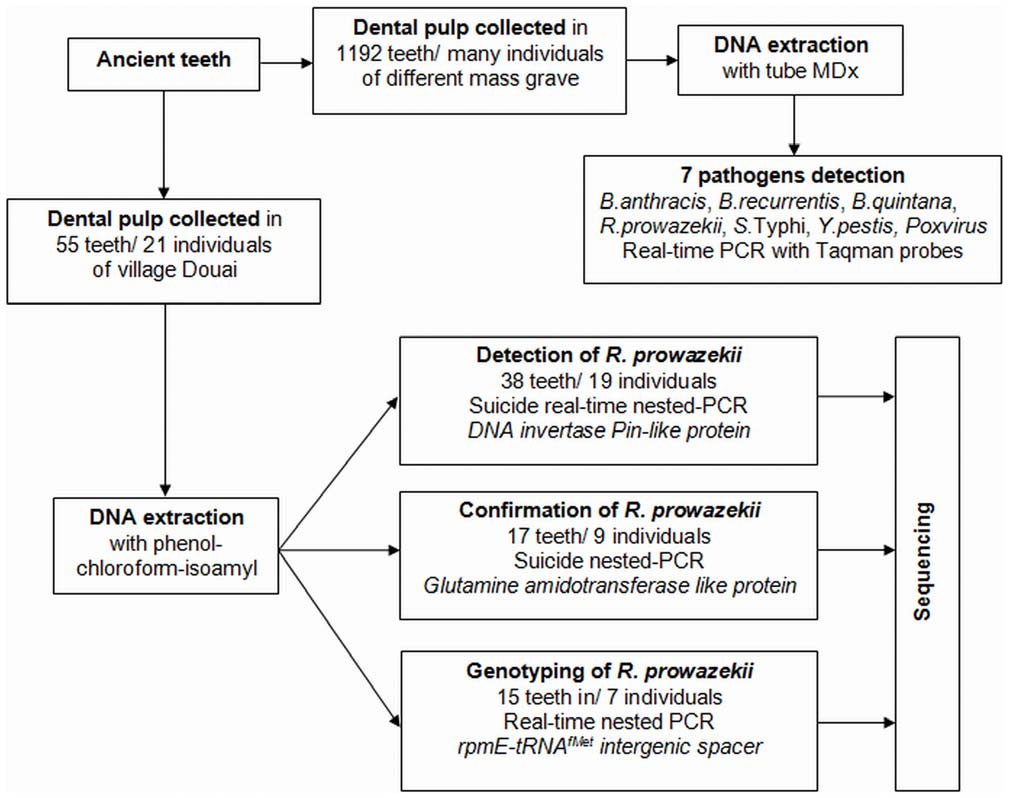
Summary of the materials and methods used in this study.

Douai is a village located in northern France. It was disputed between France and the Great Alliance of La Haye between 1702 and 1712 [9], [10]. The site of the street Martin-du-Nord was discovered during building construction in 1981 (Figure 2). A total of twelve multiple burials and four individual graves have been uncovered at this site. The graves appeared to be scattered over the plot in various directions (Figure 3), and the individuals were deposited head-to-foot into exiguous pits, a characteristic of disaster graves linked to sudden and massive mortality. This type of burial can also occur during an epidemic [11], [12]. The demographic profile of young males as well as historical documents suggested a military installation. Five individuals with evidence of traumatic injuries were buried in a single pit. The cause of death could not be attributed to trauma in the other individuals, and 21 such individuals were studied herein, from whom a total of 55 teeth were used for the molecular investigations [13], [14] (Figure 4).

### High-throughput detection of pathogens

Dental pulp recovered as previously described [4] was incubated overnight at 56°C with 600 µL ATL buffer and 50 µL proteinase K. DNA was then extracted by using QIAamp Media MDx Bacterio-pulverisation in the BioRobot® MDx workstation in a final 100 µL volume (Qiagen GmbH, Hilden, Germany). Seven PCR primer pairs and seven probes were designed for the specific detection of *Bacillus anthracis* (anthrax), *Borrelia recurrentis* (louse-borne relapsing fever), *Bartonella quintana* (trench fever), *Rickettsia prowazekii* (epidemic typhus), *Salmonella enterica* Typhi (typhoid fever), *Poxvirus* (smallpox) and *Yersinia pestis* (plague) (Table 1). Real-time PCR amplification was performed using the QuantiTech Probe PCR Kit (Qiagen) and a 7900HT Fast Real-Time PCR System (Applied Biosystem, Courtaboeuf, France). Each well of a 384-well plate was filled with 10 µL Mix Quantitech, 2 µL sterile water, 2 µL of 2 pmol/µL probe, 0.5 µL forward primer (10 pmol/µL), 0.5 µL reverse primer (10 pmol/µL) and 5 µL DNA. Amplification consisted of 15-min activation at 95°C followed by 50 cycles of 30-sec denaturation at 95°C and 1-min hybridisation at 60°C. In every plate, two wells containing sterile water and two wells containing DNA extracted from dental pulp collected from skeletons devoid of anthropologic evidence of infection were used as negative controls.

**Table 1:** Primers for molecular detection of all pathogens into 1192 acient teeth.

Desired specificity	Gene	Name	Probe sonde and primers	Sequence
*Bacillus anthracis*(anthrax)	pag	Bant_pag_P	6 FAM- TAC CGC AAA TTC AAG AAA CAA CTG C -TAMRA	94 bp
		Bant_pag_F	5′- AGG CTC GAA CTG GAG TGA A -3′	
		Bant_pag_R	5′- CCG CCT TTC TAC CAG ATT T -3′	
*Borrelia recurrentis*(louse-borne relapsing fever)	unknown	Brec_P	6 FAM- CTG CTG CTC CTT TAA CCA CAG GAG CA -TAMRA	111 bp
		Brec_F	5′- TCA ACT GTT TTT CTT ATT GCC ACA -3′	
		Brec_R	5′- TCC TTA TGT TGG TTA TGG GAT TGA -3′	
*Bartonella quintana*(Trench fever)	ITS	Barto ITS_P	6 FAM- GCG CGC GCT TGA TAA GCG TG -TAMRA	102 bp
		Barto ITS_F	5′- GAT GCC GGG GAA GGT TTT C -3′	
		Barto ITS_R	5′- GCC TGG GAG GAC TTG AAC CT -3′	
*Rickettsia prowazekii*(typhus)	ompB	Rpr_ompB_P	6 FAM- CGG TGG TGT TAA TGC TGC GTT ACA ACA -TAMRA	134 bp
		Rpr_ompB_F	5′- AAT GCT CTT GCA GCT GGT TCT -3′	
		Rpr_ompB_R	5′- TCG AGT GCT AAT ATT TTT GAA GCA -3′	
*Salmonella Typhi*(typhoid fever)	unknown	Styp_put_P	6 FAM- GCT TTT TGT GAA GCA ACG CTG GCA -TAMRA	138 bp
		Styp_put_F	5′- CTC CAT GCT GCG ACC TCA AA -3′	
		Styp_put_R	5′- TTC ATC CTG GTC CGG TGT CT -3′	
*Poxvirus*(smallpox)	HA	Var_HA_P	6 FAM- AAG ATC ATA CAG TCA CAG ACA CTG T -TAMRA	100 bp
		Var_HA_F	5′- GAC KTC SGG ACC AAT TAC TA -3′	
		Var_HA_R	5′- TTG ATT TAG TAG TGA CAA TTT CA -3′	
*Yersinia pestis*(plague)	pla	Yper_PLA_P	6 FAM- TCC CGA AAG GAG TGC GGG TAA TAG G -TAMRA	98 bp
		Yper_PLA_F	5′- ATG GAG CTT ATA CCG GAA AC -3′	
		Yper_PLA_R	5′- GCG ATA CTG GCC TGC AAG -3′	

### Suicide PCR detection of *R. prowazekii*

The Douai specimens were further examined with a suicide nested PCR protocol after conventional phenol chloroform DNA extract [4]. The program PerlPrimer version 1.1.6 was used to design PCR primers. The first suicide nested-PCR targeted a 206-base pair fragment of the *Rickettsia prowazekii* DNA invertase Pin-like protein (ORF0698; gi|3861237|emb|AJ235273.1|) by combining external primers RpDet-F1 5′-GTTGGATATATAAGGGTTTC-3′ and RpDet-R2 5′-CCGAGTCTATCTAATTTCCA-3′ and internal primers RpDet-F3 5′-ATGATCGTCAAGTGTTCGAT-3′ and RpDet-R4 5′-TAGACAGTCGCCATCTTGTA-3′; the final expected PCR product was 152 bp (Table 2). This region had never been amplified in our laboratory. The first round PCR mix contained 1.6 µL MgCl_2_, 0.5 µL bovine serum albumin (BSA), 2 µL Master Mix (Light Cycle® FastStart DNA Master Sybr Green, Roche Applied Science, France), 1 µL of a 10 µM solution of RpDet-F1, 1 µL of a 10 µM solution of RpDet-R2, 8.9 µL sterile water and 5 µL DNA (experimental tube) or 5 µL sterile water (negative control tube). The first round of amplification was done using a LightCycler™ apparatus (Roche Diagnostics) and the following conditions: 10-min activation at 95°C followed by 45 cycles of 15-sec denaturation at 95°C, 20-sec hybridisation at 55°C and 25-sec elongation at 72°C. PCR products were recovered by centrifugation of capillaries at 380 g for 2 minutes. The nested PCR was performed in the capillary containing 1.6 µL MgCl_2_, 0.5 µL BSA, 2 µL Master Mix, 1 µL of 10 µM solution of RpDet-F3; 1 µL of 10 µM solution of RpDet-R4; 8.9 µL sterile water and 5 µL first round PCR product. The second amplification in the LightCycler™ apparatus used the following conditions: activation at 95°C for 10 minutes and 45 cycles of denaturation at 95°C for 15 seconds, hybridisation at 57°C for 20 seconds, elongation at 72°C for 25 seconds. Nested PCR products were detected on a 2% agarose gel (Invitrogen™, Paisley, Scotland) in the presence of molecular weight marker VI (Boehringer Mannheim, Germany).

**Table 2:** Primers for detection and genotyping of *R. prowazekii* into ancient teeth of Douai.

	PCR	Name	Primers	Tm	Sequence zise	Reference
Detection of*R. prowazekii*	Real-time PCR	RpDet-F1RpDet-R2	5′-GTTGGATATATAAGGGTTTC-3′ 5′-CCGAGTCTATCTAATTTCCA-3 ′	55°C	206 bp	rpr_ORF0698 site-specific recombinases, DNA invertase Pin-like protein
	Nested PCR	RpDet-F3RpDet-R4	5′-ATGATCGTCAAGTGTTCGAT-3 ′ 5′-TAGACAGTCGCCATCTTGTA-3′	57°C	152 bp	
Confirmation of*R. prowazekii*	PCR standard	Rpro-F1Rpro-R1	5′-ACTGTTATTACCGATCTTGCCA-3′ 5′-TGGTTGATGCTAGGTTATTTGG-3′	58°C	187 bp	rpr_ORF0700, glutamine amidotransferase-like protein
	Nested PCR	Rpro-F11Rpro-R11	5′-GTATTAAGAATTTGATGCCACCA-3′ 5′-GTTATTAGTCCAAATGACGTGAA-3′	62°C	130 bp	
*R. prowazekii*genotyping	Real-time PCR	rpmE-F1rpmE-R2	5′-CCGGAAATGTAGTAAATCAATC-3′ 5′-CTGAGAATTTAAAGATTTATCTG-3 ′	59°C	210 bp	Yong Zhu et al., 2005 rpmE-tRNA^fMet^ intergenic spacer
	Nested PCR	rpmE-F3rpmE-R4	5′-CTTTCGATAGCAAGAAAGAAGC-3 ′ 5′-CAGAGTATTAGTAGACGATACG 3′	62°C	115 bp	

A second suicide nested-PCR targeted a 187-base pair fragment of the *R. prowazekii* glutamine amidotransferase-like protein (rpr_ORF0700; gi|3861237|emb|AJ235273.1|) by combining external primers Rpro-F1 5′-ACTGTTATTACCGATCTTGCCA-3′ and Rpro-R1 5′-TGGTTGATGCTAGGTTATTTGG-3′ and internal primers Rpro-F11 5′-GTATTAAGAATTTGATGCCACCA-3′and Rpro-R11 5′-GTTATTAGTCCAAATGACGTGAA-3′; the final expected PCR product was 130 bp (Table 2). This region had never been amplified in our laboratory. The first round of PCR was performed using a HotSartTaq DNA Polymerase Kit (Qiagen) with 0.8 µL MgCl_2_, 0.2 µL HotStart Taq, 2.5 µL 10X PCR buffer, 2.5 µL dNTP, 0.5 µL BSA, 0.5 µL of a 10 µM solution of each Rpro-F1 and Rpro-R1, 12.5 µL sterile water and 5 µL DNA (experimental tube) or 5 µL sterile water (negative control tube). The first round of amplification was done in an ABI GeneAmp™ 2700 thermocycler (Applied Biosystems, CA, USA) under the following conditions: 10-min activation at 95°C and 45 cycles of 30-sec denaturation at 95°C, 45-sec hybridisation at 58°C and 90-sec elongation at 72°C. The PCR products were purified using the Millipore plate protocol and suspended into 40 µL water. The second round of PCR was performed using 0.8 µL MgCl_2_, 0.2 µL HotStart Taq, 2.5 µL 10X PCR buffer, 2.5 µL dNTP, 0.5 µL BSA, 0.5 µL Rpro-F11, 0.5 µL Rpro-R11, 12.5 µL sterile water and 5 µL first round PCR product. The second amplification was performed using the following conditions: 10-min activation at 95°C and 45 cycles of 30-sec denaturation at 95°C, 45-sec hybridisation at 62°C and 90-sec elongation at 72°C. Nested PCR products were detected on a 2% agarose gel (Invitrogen™, Paisley, Scotland) in the presence molecular weight marker VI (Boehringer Mannheim, Germany).

Nested PCR products were purified using the Millipore plate protocol and suspended in 50 µL water. The sequencing reaction was carried out in a tube containing 3 µL Big Dye Terminator, 0.5 µL forward or reverse primer, 3.5 µL purified PCR product and 3 µL sterile water using the following conditions: activation at 95°C for 5 minutes followed by 25 cycles consisting of 30-sec denaturation at 96°C, 20-sec hybridisation at 55°C, 4-min elongation at 60°C and 7-min extension at 15°C. The sequencing products were purified on Séphadex® G50 5% gel and analysed with the ABI PRISM 3100 Genetic Analyser (HITACHI). The sequences were read and corrected by using the software ChromasPro version 1.34 and then aligned with BLAST to compare the sequences available in GenBank.

### Genotyping of R. prowazekii

The software PerlPrimer version 1.1.6 was used to design two pairs of primers targeting a 210-bp sequence (external primers: rpmE-F1 5′-CCGGAAATGTAGTAAATCAATC-3′ and rpmE-R2 5′-CTGAGAATTTAAAGATTTATCTG-3′) and a 115-bp sequence (internal primes: rpmE-F3 5′-CTTTCGATAGCAAGAAAGAAGC-3′ and rpmE-R4 5′-CAGAGTATTAGTAGACGATACG 3′) on the rpmE-tRNA^fMet^ intergenic spacer (gi|56967982|gb|AY695449.1|) (Yong Zhu et al., 2005) (Table 2). Genotyping was performed by mixing 1.6 µL MgCl_2_, 0.5 µL BSA, 2 µL Master Mix, 1 µL of 10 µM solution of rpmE-F1; 1 µL of 10 µM solution of rpmE-R2, 8.9 µL sterile water and 5 µL DNA (experimental tube) or 5 µL sterile water (negative control tube) in a Stratagene plate. The first round of amplification was performed in a Stratagene™ thermocycler (Agilent Technologies Company) using the following conditions: activation at 95°C for 10 minutes, 45 cycles of denaturation at 95°C for 30 seconds, hybridisation at 59°C for 30 seconds, elongation at 72° C for 1 minute. Nested PCR was realised in a Stratagene plate containing 1.6 µL MgCl_2_, 0.5 µL BSA, 2 µL Master Mix, 1 µL of 10 µM solution of rpmE-F3, 1 µL of 10 µM solution of rpmE-R4, 8.9 µL sterile water and 5 µL first round PCR product. The second amplification in the Stratagene™ thermocycler was performed using the following conditions: activation at 95°C for 10 minutes followed by 45 cycles consisting of denaturation at 95°C for 30 seconds, hybridisation at 62°C for 30 seconds and elongation at 72°C for 1 minute. The nested PCR products were detected on a 2% agarose gel (Invitrogen™) in the presence of molecular weight marker VI (Boehringer Mannheim). The PCR products were sequenced as described above.

### Prevention of DNA contamination

all manipulations of ancient materials were done in two successive laboratories where *R. prowazekii* had never been previously amplified. Each step was conducted in a separate room under a hood with air-capture. All instruments were sterilised and used only once. No positive control was included, and one negative control consisting of sterile water was used for each three or five samples.

## Results

### High-throughput detection of pathogens

A total of 1.192 dental pulp specimens collected from several burial sites in France were analysed by high throughput detection, including 40 specimens collected in Douai tested blindly. Whereas the negative controls remained negative, high throughput real-time PCR detected *B. quintana* DNA in 1/40 dental pulp specimens, and no other pathogen was detected in these 40 specimens. We then attempted to detect *R. prowazekii* using a more sensitive technique.

### Molecular detection of *R. prowazekii*

**Figure 5:**
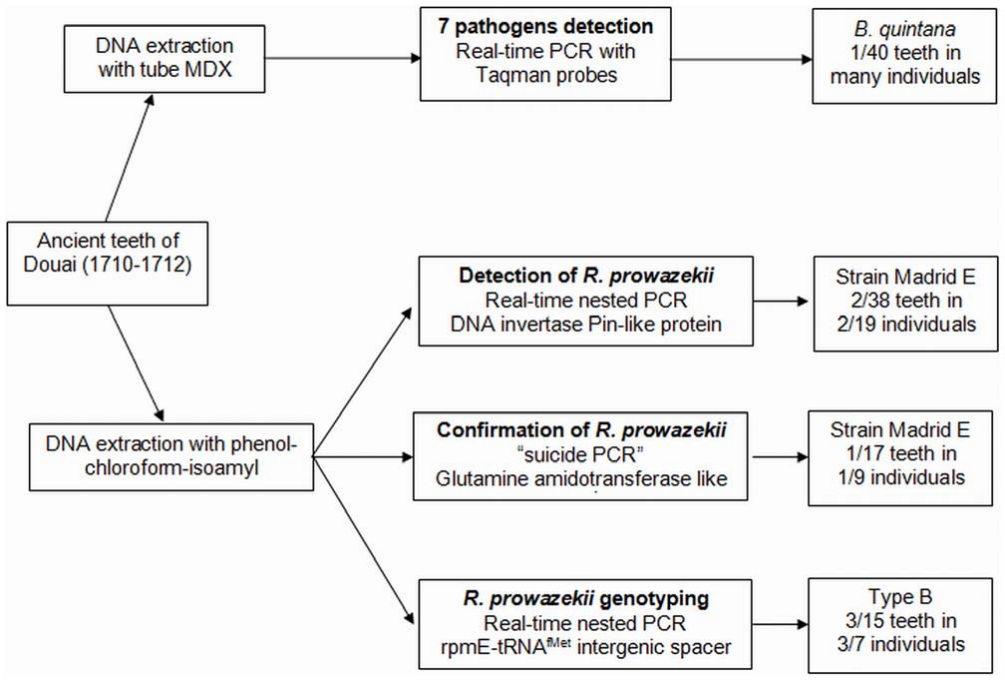
Summary of *R. prowazekii* detection and genotyping results.

In all of the experiments, the negative controls remained negative. The first suicide real-time nested PCR detected *R. prowazekii* DNA in 2/38 (5.3%) ancient teeth collected from 2/19 (10.5%) different individuals (coded as A6-4 and A10-19). Sequence alignment yielded 100% sequence similarity with the reference *R. prowazekii* strain Madrid E (Genbank accession emb|AJ235273.1|RPXX04). As for the second suicide PCR, it detected *R. prowazekii* DNA in 1/17 (5.9%) dental pulp specimens collected from 1/9 (11%) different individuals (coded as 126/220-39) (Figure 5).

### Genotyping of *R. prowazekii*

**Figure 6:**
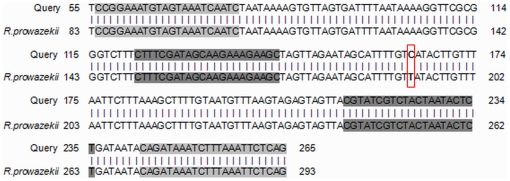
Result of Genbank alignments for genotyping. Grey boxes indicate the locations of PCR primers.

In the genotyping, the negative controls remained negative while we amplified the *R. prowazekii* rpmE/tRNA^fMet^ intergenic spacer sequence in 3/15 (20%) ancient teeth collected from 3/7 (43%) different individuals (coded as 075-24, 077-27, 1074-35). All PCR products yielded an identical sequence exhibiting 100% sequence similarity to *R. prowazekii* type B, which is characterised by a T to C substitution at position 111 (Figure 6). Merging all of the PCR results yielded positive detection of *R. prowazekii* DNA in 6/55 (11%) teeth collected from 6/21 (28.6%) individuals.

## Discussion

The results presented herein were interpreted as authentic. Indeed, we selected teeth that had a closed apex and that were free of dental caries and traumatic lesions to minimise any risk of external contamination of the dental pulp. For the first time, we used a high throughput paleomicrobiological approach to screen 1192 teeth for seven infectious agents (8,344 tests). This technique is less sensitive than suicide PCR but is well adapted to testing numerous samples. Our work was carried out in a laboratory where *R. prowazekii* had never been worked on, nor had *R. prowazekii* DNA been extracted. All operations were carried out under a hood with air-capture using sterilised instruments that were used only once. All the negative controls remained negative. We obtained an original sequence that was consistently found in several teeth collected in several individuals, its uniqueness thus giving much confidence. After blindly detecting *B. quintana* in one tooth from Douai, we decided to employ more sensitive techniques to recover *R. prowazekii* from other teeth from this site.

In this study, we observed that not all dental pulp specimens collected from a single individual yielded a positive PCR product. Such variability in the positivity of PCR-based detection has been previously observed in detecting *Yersinia pestis* DNA [4]. Specific *R. prowazekii* sequences were detected in 6/55 (11%) teeth collected in 6/21 (28.6%) individuals; this prevalence is significantly higher (*P*<0.05, test χ^2^) than that previously reported in the Napoleon's Grand Army study, which detected *R. prowazekii* in 4/72 (5.6%) teeth collected from 3/35 (8.6%) soldiers [15]. As we also found an individual infected by *B. quintana*, we suspect the circulation of louse-borne diseases in this place, but we did not recover *Borrelia recurrentis* DNA.

In the present study, we also took advantage of the dental pulp to detect and genotype 300-year-old *R. prowazekii* DNA. Dental pulp is a specialised conjunctive tissue occupying the central position in the tooth, where it is protected from the external environment by the dentine. We previously demonstrated that blood-borne bacteria could be detected in dental pulp both by culture and by molecular detection of specific DNA sequences [16]–[18]. In addition, dental pulp is the only conjunctive tissue that can persist for several thousand years after the degradation of other tissues because it is very well preserved within the dentine and enamel, which are the hardest tissues of the human body [4]. In 2004, Tran H Lam et al. showed that PCR amplifying a fragment of 286 base pairs of the 16S rRNA gene could identify several bacteria in the pulp of ancient teeth dated to the 17th century [19]. It is difficult to amplify fragments of DNA more than 300 base pairs from old samples [4], [6]. For these reasons, we chose a fragment of 115–210 base pairs for amplification. Nested PCR, which is effected by two successive PCR reactions with two pairs of external-internal primers, can increase PCR's sensitivity and specificity considerably. With the combination of the benefits of real-time PCR [20] and nested PCR, we have successfully amplified an ancient bacterial DNA sequence from the 18th century and identified it as *R. prowazekii* genotype B. It was later shown that real-time PCR is a rapid, specific and sensitive method for detecting *R. prowazekii* in blood [20], [21]. Sequence analysis of the rpmE/tRNA^fMet^ spacer in 15 modern *R. prowazekii* DNA samples found three genotypes: A, B and C. Genotype B had been sequenced in the avirulent *R. prowazekii* Madrid E strain (derived from a Spanish isolate [22]), in its virulent revertant *R. prowazekii* Evir in two blood isolates from Russia and Algeria, and in four lice collected in Rwanda and Burundi [23]. We herein demonstrated that this genotype was already present in 18^th^-century Europe.

Despite the fact that typhus epidemics has been depicted in historical sources for two millennia or so [24], only few demonstrations of *R. prowazekii* issued from ancient materials have been provided. One individual diagnosed with fatal epidemic typhus in Maryland in 1901 was demonstrated by using immunohistological detection ninety years later to have died of Rocky Mountain spotted fever caused by *Rickettsia rickettsii* [25]. In another report, sequence-based analysis of remains of Napoleon's Grand Army soldiers yielded molecular evidence for *R. prowazekii* and *B. quintana* in an estimated one-third of the individuals [26]. The data herein reported remind us that epidemic typhus, a disease now confined to relatively limited geographic areas, had a broad geographic range of prevalence only a few centuries ago, being one of the plagues reported in historical descriptions (Table 3) [27]. In the past, several typhus epidemics have been described in central Africa [28]. Today, cases of typhus are still described in Peru [29] and in industrial cities of Russia [30], Algeria [31] and France [32]. Further studies applying the techniques herein described to the remains of individuals in America, Europe and Africa may help to paint a clearer picture of the evolution and spread of epidemic typhus in connection with human history.

**Table 3:** Dental pulp: a source for the paleomicrobiology of ancient epidemics.

Epidemic	Site - date	Materials and Methods	Results	Reference
Plague	Lambesc – 1590 and Marseille – 1722 (France)	Dental pulp, DNA amplification by PCR, gene RpoB (133-bp) and gene pla (300-bp)	*Y. pestis* detected in 6/12 teeth	[3]
	Saint-Côme and Saint-Damien (Montpellier) – 14^th^ century (France)	Dental pulp, “suicide” PCR, gene pla (148-bp)	*Y. pestis* detected in 20/23 teeth	[5]
	Sens: 5^th^–6^th^ century, Dreux: 12^th^–14^th^ century and Monpellier – 1348 (France)	Dental pulp, DNA amplification by PCR, spacer YP	*Y. pestis* strain Orientalis detected in 7/11 individuals	[31]
	Aschheim – 6^th^ century (Upper Bavaria)	Dental pulp, “suicide” PCR, gene pla (148-bp)	*Y. pestis* detected in 2/6 teeth	[32]
	Vienne: 7th–9th century, Martigues: 1720–1721 and Marseille – 1722	Dental pulp, suicide-nested PCR, gene glpD (191-bp)	*Y. pestis* strain Orientalis detected in 5/46 teeth	[33]
	Lambesc – 1590, Saint-Pierre: 1628–1632, Draguignan: 1649–1650, Martigues: 1720–1721, Berre l'Etang: 1720–1721, Marseille – 1722 (France)	Dental pulp and spongy bone, immuno-detection by RDT, F1 antigen	*Y. pestis* detected in 19/28 individuals	[34]
Typhoid fever	Athens: 430–426 BC (Greek)	Dental pulp, “suicide” PCR, gene osmC-clyA (322-bp) and gene narC (360-bp)	*S. enterica* Typhi detected in 3/3 teeth	[35]
Rocky Mountainspotted fever	Maryland – 1901 (USA)	Immunohistology detection	Detection of *R. rickettsii*	[22]
Cat-cratch disease	Compiègne – 16^th^ century, Montbéliard – 14^th^ century and Paris – 13^th^ century	Dental pulp of cats, nested PCR, gene groEL (269 – bp) and gene Pap31 (164 – bp)	*B. henselae* detected in 3/135 teeth of cats	[36]
Trench fever	Roaix: 2100–2200 BC and Peyraoutes: 2230–1950 BC (France)	Dental pulp, nested PCR, gene hemin-binding protein-E (283-bp) and gene groEL (269-bp)	*B. quintana* detected in 1/12 teeth	[6]
Typhus and trench fever	Vilnius – 1812 (Lithuania)	Lice and dental pulp, DNA amplification by PCR, gene dnaA (141–279 bp) and gene hbpE (282–429 bp)	*R. prowazekii* detected in 4/72 teeth, *B. quintana* detected in 7/72 teeth and 3/5 lice	[7]
Typhus and trench fever	Douai: 1710–1712 (France)	Dental pulp, real-time PCR and “suicide PCR”, gene ITS (102-bp) and gene DNA invertase Pin-like protein (152–206 bp), gene glutamine amidotransferase-like protein (130–187 bp), rpmE-tRNA^fMet^ intergenic spacer (115-210 bp)	*B. quintana* detected in 1/40 teeth and *R. prowazekii* strain Madrid E type B detected in 6/55 teeth	Present work

Taken together, the results reported herein show clearly that an epidemic disease transmitted by lice prevailed during the long siege of Douai. Diseases transmitted by body lice were probably extremely frequent in the past. When sanitary arrangements are degraded, lice are likely to quickly expand and the population of lice can increase by 10% per day [33] when the conditions of hygiene are met [29]. Under these conditions, it is easy to imagine a *B. quintana* epidemic persisting in Europe for an extremely long time. The oldest trace of infection of humans by this bacterium goes back 4.000 years [34]. Epidemic typhus appeared in Europe later. The majority of authors suggest that it was introduced by the Spanish returning from America [35]. The war in Douai was that of the Spanish succession, and one can propose that it was imported by Spanish soldiers. It is interesting therefore to observe that the genotype B found here is identical to that of a Spanish isolate from the beginning of the XX^th^ century in Spain. The first descriptions go back to Fracastor, and early in the 16th century a number of epidemics compatible with the diagnosis of typhus were reported. However, at the beginning of the 18th century, the clinical individualisation of epidemic typhus, like that of trench fever of the trenches, was not yet carried out. The first definition of typhus was provided by Boissier de Sauvages in 1772 [36].

In conclusion, the molecular diagnosis of past epidemic infections related to the teeth made it possible to identify the first outbreak of epidemic typhus in the 18th century in the context of a pan-European Great War. Working together, molecular biologists, dentists and anthropologists have explored burials from catastrophes to identify the prevailing epidemics in past centuries.
